# Association between biomass fuel use and the risk of cognitive impairment among older populations in China: a population-based cohort study

**DOI:** 10.1186/s12940-021-00706-1

**Published:** 2021-02-24

**Authors:** Min Du, Liyuan Tao, Lin Zhu, Jue Liu

**Affiliations:** 1 Department of Epidemiology and Biostatistics, School of Public Health, Peking University, No.38, Xueyuan Road, Haidian District, Beijing, 100191 China; 2 Research Center of Clinical Epidemiology, Peking University Third Hospital, No.49 Huayuan North Road, Haidian District, Beijing, 100083 China; 3 Center for Primary Care & Outcomes Research, School of Medicine, Center for Health Policy, Freeman Spogli Institute for International Studies, Stanford University, 450 Jane Stanford Way, Stanford, CA 94305–2004 USA

**Keywords:** Biomass fuel, Cognitive impairment, Chinese, Elderly, Cohort study

## Abstract

**Background:**

Cohort studies on the impact of biomass fuel use for cooking on cognitive impairment among older population are still lacking in China and elsewhere. The aim of this study was to examine whether biomass fuel use for cooking is associated with cognitive impairment in Chinese adults aged 65 years or older.

**Methods:**

The prospective population-based cohort study of the Chinese Longitudinal Healthy Longevity Survey (CLHLS) included participants aged 65 years or older in 2014 who were followed-up until 2018 in 23 provinces in China. The Mini-Mental State Examination (MMSE) was used to assess cognitive function, and cognitive impairment was defined as total MMSE scores less than 18. The association between biomass fuel use and cognitive impairment was evaluated using the Cox proportional hazards model.

**Results:**

Of the 4145 participants included at baseline, participants who reported that they used biomass fuel for cooking (40.43%; IR: 3.11 versus 2.77 per 100 person-years; aHR: 1.27, 95% CI: 1.02–1.58) had a higher risk of cognitive impairment compared with participants who used clean fuels (53.75%). A stratified analyses showed greater effect estimates of cognitive impairment in the older people that lived in the rural areas (aHR: 1.444, 95% CI: 1.08–3.90) and never smoked (aHR: 1.33, 95% CI: 1.04–1.71).

**Conclusions:**

These findings demonstrated that biomass fuel used for cooking was associated with cognitive impairment, as defined by MMSE, in a population-based study of elderly in China. To prevent cognitive impairment, the structure of cooking fuels requires improvements.

**Supplementary Information:**

The online version contains supplementary material available at 10.1186/s12940-021-00706-1.

## Background

Although clean fuels for cooking have been recommended in recent years, globally, 80% of the rural population and < 15% of the urban population still lack access to clean cooking [1]. Furthermore, biomass fuel use has been linked to household air pollution (HAP) with a higher component of particulate matter (PM) compared with the combination of biomass & liquefied petroleum gas (LPG) and LPG [2]. Studies have reported that particulate pollution from kitchens was higher when people used biomass fuels, including charcoal, wood, and grass, compared with clean fuels, including electricity and liquefied petroleum gas [3, 4]. HAP from the burning of biomass fuels affects approximately three billion individuals worldwide [1, 5]. Importantly, older people’s frail health status may be easily affected by HAP. According to the 2016 global burden disease study, lower respiratory infections caused 1,080,958 deaths among adults older than 70 years old in 2016, and HAP may have been the primary cause [6]. Many studies have revealed that HAP not only had an effect on respiratory diseases including breathlessness, asthma, and even lung cancer [7], but also heart health, including elevated blood pressures and heart rates among elderly people [8]. Recently, one study reported that solid fuels used for cooking significantly increased the possibility and exacerbation of chronic lung diseases and heart diseases of the elderly in rural China [9]. Thus, the burning of biomass fuels on the health outcomes of older people requires more attention due to the higher HAP levels.

Cognitive impairment means that a person has problems with remembering, learning new things, concentrating, or making decisions, which all affect quality of life [10]. With rapid population aging, one national study estimated that the prevalence of cognitive impairment was 9% among Chinese older persons in 2011, which was higher than most countries of the world [11]. Growing evidence suggests that air pollution has an negative effect on cognition among older adults. In addition, studies have primarily explored the association between HAP and respiratory and cardiovascular disease among older adults, but the effects of indoor combustion of biomass fuel for cooking on the central nervous system have not been broadly recognized among older adults. A cross-sectional study found that the combustion of wood or coal fuels was associated with poorer cognitive performance after adjusting for demographic, household, health, and economic characteristics among adults aged over 50 years old in Mexico [12]. Cao et al. found that solid cooking fuel use was associated with a greater decline in cognitive score overall, especially for episodic memory, according to follow-up studies that included 8397 middle-aged and older participants [13]. However, a prospective cohort study on the association of biomass fuel use for cooking with cognitive impairment among Chinese older people was limited.

Biomass fuel use for cooking is the primary source of HAP, however, the role of biomass fuel use in cognitive impairment has not been well elaborated. A large multicenter cohort study of the association between biomass fuel use and cognition in older adults is required for earlier intervention to improve mental health. Moreover, the current available studies did not control for other confounders, including exercise [14], diet [15], and other factors that could have an effect on cognition. Based on previous theory and empirical research, the aim of this study was to assess the association between biomass fuel use for cooking and the risk of cognition impairment among Chinese elderly people in a nationwide prospective cohort study.

## Methods

### Study design and participants

In this study, data obtained from the Chinese Longitudinal Healthy Longevity Survey (CLHLS), which is an ongoing, prospective cohort study that covered 23 out of 31 provinces in China, was analyzed. This study was established in 1998, with subsequent follow-up and recruitment of new participants in 2000, 2002, 2005, 2008, 2011, 2014, and 2018. Information regarding the health and life situations of individuals aged 65 and older were provided in the CLHLS, and more details of the study have been described elsewhere [16]. The present analysis included data from the seventh wave of the CLHLS in 2014 (at baseline), which included the question “Which fuels are normally used for cooking in your home?” The follow-up survey was conducted in 2018. The CLHLS was approved by the Ethical Review Committee of Peking University (IRB00001052–13074). All of the participants signed an informed consent at the time of participation. The research was performed in accordance with the Declaration of Helsinki.

**Fig. 1:**
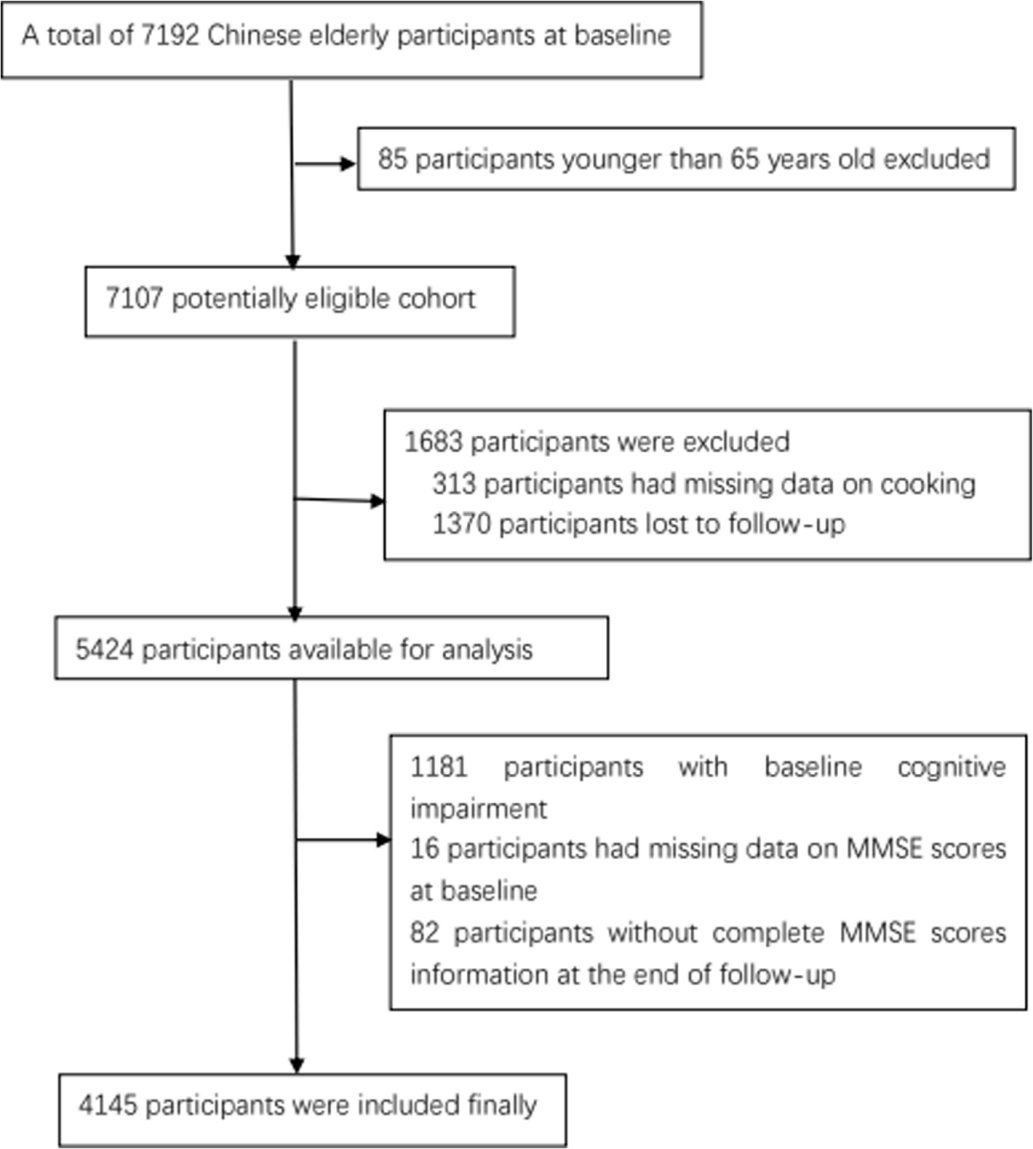
The full inclusion and exclusion process of the research

The 2014 survey wave included 7192 Chinese elderly individuals. Participants that were excluded were 85 participants that were younger than 65 years old, 313 participants that were missing data on cooking fuels, 1370 participants that were lost to follow-up in 2018, 1181 participants that had baseline cognitive impairments, and 16 participants with missing data on the baseline MMSE scores. Furthermore, 82 participants were excluded that did not have complete MMSE score information upon the first follow-up survey. Hence, a total of 4145 participants were finally included. Figure 1 shows the full inclusion and exclusion process of the research participants in this study.

### Assessment of biomass fuel use for cooking

The participant’s household exposure to cooking fuels was assessed using a questionnaire that was answered at baseline by the participants that contained the question “Which fuels are normally used for cooking in your home?” The cooking fuels were categorized as biomass fuels (charcoal, firewood/straw), clean fuels (electricity, gas, solar energy), never cooked in the home, and others (e.g., fuel oil, coal, and others).

### Outcome

The CLHLS participants’ cognitive function was measured using the widely used screening tool called the Chinese version of the Mini-Mental State Examination (MMSE) that consisted of 11 questions covering orientation, registration, attention, calculation ability, recall, and language ability. This tool was used in 2014 and 2018 at two time points [17]. Several items on the Chinese version of the MMSE were changed based the cultural contexts of China, and the validity and reliability was still good [18]. All of questions were answered by the respondents without a proxy. The total MMSE scores ranged from 0 to 30, and cognitive impairment was defined as a total MMSE score less than 18, whereas a participant with a score of 18 or higher was classified as having no cognitive impairment [18, 19].

### Covariates

We attempted to examine as many factors as possible that have been found to be associated with fuel use for cooking and cognitive impairment [14, 15]. Trained investigators collected information, including demographic characteristics, lifestyle habits, and health status, using a standardized questionnaire. All of the surveys were face-to-face interviews conducted at the participant’s home. If participants were illiterate, investigators helped them to complete the questionnaire. Details of the sample design had been described elsewhere, and the data quality was reported to be generally good [20]. Demographic characteristics included age (65–74, 75–84, and ≥ 85 years old), sex (male/female), education (no school/1 year or more), residence (urban/rural), household income (< 10,000/10001–30,000/> 30,000 yuan), and marital status (unmarried/married/divorced/widowed). Lifestyle habits included smoking status (non-smoker/smoker), drinking status (non-drinker/drinker), regular exercise (yes/no), ventilation of the kitchen when cooking at home (no ventilation/kitchen ventilation or fan/opening a window/unknown), and diet of fresh fruit (almost or quite often/occasionally/rarely or never) and vegetables (almost or quite often/occasionally/ rarely or never). The health status included limited in activities because of health problems during the last six months (yes/no), body mass index (BMI, weight/height^2^, kg/m2) (underweight (< 18.5)/normal (18.5–24.9)/overweight (25–29.9)/obese (≥30)/unknown), and self-reported previous diseases including hypertension (yes/no/unknown), diabetes (yes/no/unknown), heart diseases (yes/no/unknown), and stroke (yes/no/unknown).

The weights and standing heights were measured directly by trained investigators. The BMI was categorized according to the cutoff points of the Working Group on Obesity in China [21]. The remaining variables were directly collected using the standardized questionnaire at baseline, except ventilation of the kitchen when cooking at home, which was collected only during the 2018 follow-up survey.

### Data analysis

Baseline characteristics of the study population were described as the means ± standard deviations (SDs) for the continuous variables or percentages for the categorical variables according to cooking fuel exposure. Time to cognitive impairment (event = 1) was defined as the period from baseline to the earlier assessment of the MMSE. For those who died without information on cognitive impairment (event = 2), the time from baseline to time of death was calculated. Censored (event = 0) observations were defined as participants who did not have cognitive impairment, and the censoring time was calculated from baseline to the last assessment of the MMSE. Cox proportional hazards models were used to assess the association of biomass fuel use with cognitive impairment. The follow-up ended on the date of death or the end of the study, whichever was earlier. A sensitivity analysis was performed by fitting the different models to examine the robustness of the estimation. Model 1 was a univariate model. Basic demographic characteristics were added in model 2, including age, sex, education, residence, household income, and marital status. All of the covariates in model 3 were adjusted by adding the smoking status, drinking status, regular exercise, diet of fresh fruit and vegetables, limited in activities because of health problems during the last six months, BMI, and self-reported previous diseases based on model 2. A stratified analyses was performed by sex, education, residence, household income, smoking status, drinking status, fresh fruit, limited in activities because of health problems during the last six months and self-reported previous diseases, and the significance of interaction was tested by including a two-way interaction term in the final model. In addition, in order to test the robustness of the results: 1) Cox models for the excluded 3047 participants were performed (Supplemental Table 1); and 2) Cox models were performed for the 2933 participants in the follow-up survey after 1212 participants who died were excluded (Supplemental Table 2). A *P* value less than 0.05 was significant. The crude incidence rates (IR) (per 100 person-years) of cognitive impairment across the categories of cooking fuels were calculated. The results were presented as pooled hazard ratios (HRs) with 95% confidence intervals (95% CIs). All of the analyses were performed using SPSS 26.0 and R 3.4.0.

## Results

### Basic characteristics of the participants

The characteristics of the 4145 participants without cognitive impairment at baseline (Table 1) had a mean (SD) age of approximately 82.69 (± 8.90) years old, and 51.22% of the participants were men.

**Table 1:** Characteristics of the study participants according to cooking fuel used at the baseline

Characteristics	N	Cooking fuel				χ2	*P*
		Clean fuel	Never cooked in the home	Biomass fuel	others		
Total	4145	2228 (53.75)	59 (1.42)	1676 (40.43)	182 (4.39)		
**Demographic characteristics**							
Age (years)						56.798^a^	< 0.0001
65–84	2460	1308 (53.17)	10 (0.41)	1042 (42.36)	100 (4.07)		
85–104	1606	873 (54.36)	45 (2.80)	611 (38.04)	77 (4.79)		
≥ 105	79	47 (59.49)	4 (5.06)	23 (29.11)	5 (6.33)		
Sex						10.97	0.012
Male	2123	1128 (53.13)	30 (1.41)	891 (41.97)	74 (3.49)		
Female	2022	1100 (54.40)	29 (1.43)	785 (38.82)	108 (5.34)		
Education						69.409	< 0.0001
No school	2103	998 (47.46)	39 (1.85)	960 (45.65)	106 (5.04)		
1 year or more	2040	1228 (60.20)	20 (0.98)	716 (35.10)	76 (3.73)		
Residence						325.472	< 0.0001
Urban	1815	1249 (68.82)	26 (1.43)	455 (25.07)	85 (4.68)		
Rural	2330	979 (42.02)	33 (1.42)	1221 (52.40)	97 (4.16)		
Household income CNY						486.859^a^	< 0.0001
< 10,000	1565	544 (34.76)	42 (2.68)	910 (58.15)	69 (4.41)		
10,001–30,000	1032	568 (55.04)	3 (0.29)	414 (40.12)	47 (4.55)		
> 30,000	1548	1116 (72.09)	14 (0.90)	352 (22.74)	66 (4.26)		
Marital status						83.076^a^	< 0.0001
Unmarried	34	13 (38.24)	0 (0.00)	19 (55.88)	2 (5.88)		
Married	1952	1020 (52.25)	1 (0.05)	856 (43.85)	75 (3.84)		
Divorced or widowed	2107	1168 (55.43)	57 (2.71)	781 (37.07)	101 (4.79)		
**Lifestyle habits**							
Smoking status						3.412	0.332
Non-smoker	2781	1502 (54.01)	35 (1.26)	1114 (40.06)	130 (4.67)		
Smoker	1348	717 (53.19)	23 (1.71)	557 (41.32)	51 (3.78)		
Drinking status						4.065	0.255
Non-drinker	2974	1589 (53.43)	48 (1.61)	1199 (40.32)	138 (4.64)		
Drinker	1138	623 (54.75)	11 (0.97)	461 (40.51)	43 (3.78)		
Regular exercise						253.79	< 0.0001
Yes	1383	975 (70.50)	8 (0.58)	328 (23.72)	72 (5.21)		
No	2668	1204 (45.13)	50 (1.87)	1309 (49.06)	105 (3.94)		
Ventilation of the kitchen when cooking at home						536.392^a^	< 0.0001
No ventilation	273	83 (30.40)	3 (1.10)	172 (63.00)	15 (5.49)		
Kitchen ventilation or fan	1061	845 (79.64)	9 (0.85)	182 (17.15)	25 (2.36)		
By opening window	1554	609 (39.19)	13 (0.84)	854 (54.95)	78 (5.02)		
Unknown	1257	691 (54.97)	34 (2.70)	468 (37.23)	64 (5.09)		
Fresh fruit						49.406	< 0.0001
Almost or quite often	1779	1060 (59.58)	30 (1.69)	626 (35.19)	63 (3.54)		
Occasionally	1413	711 (50.32)	13 (0.92)	620 (43.88)	69 (4.88)		
Rarely or never	943	455 (48.25)	15 (1.59)	424 (44.96)	49 (5.20)		
Vegetables						66.510^a^	0.001
Almost or quite often	3758	2052 (54.60)	52 (1.38)	1497 (39.84)	157 (4.18)		
Occasionally	289	126 (43.60)	3 (1.04)	144 (49.83)	16 (5.54)		
Rarely or never	90	48 (53.33)	3 (3.33)	31 (34.44)	8 (8.89)		
**Health status**							
Limited in activities because of health problems during the last six months						21.041	< 0.0001
Yes	3099	1637 (52.82)	33 (1.06)	1300 (41.95)	129 (4.16)		
No	1041	586 (56.29)	26 (2.50)	376 (36.12)	53 (5.09)		
Body mass index (kg/m^2^)						74.429^a^	< 0.0001
Underweight (< 18.5)	636	310 (48.74)	14 (2.20)	276 (43.40)	36 (5.66)		
Normal (18.5–23.9)	2242	1178 (52.54)	20 (0.89)	946 (42.19)	98 (4.37)		
Overweight (24–27.9)	840	493 (58.69)	11 (1.31)	308 (36.67)	28 (3.33)		
Obese (≥28)	263	165 (62.74)	4 (1.52)	75 (28.52)	19 (7.22)		
Unknown	164	82 (50.00)	10 (6.10)	71 (43.29)	1 (0.61)		
Hypertension						37.117	< 0.0001
Yes	1413	838 (59.31)	17 (1.20)	486 (34.39)	72 (5.10)		
No	2532	1283 (50.67)	37 (1.46)	1109 (43.80)	103 (4.07)		
Unknown	200	107 (53.50)	5 (2.50)	81 (40.50)	7 (3.50)		
Diabetes						26.406^a^	< 0.0001
Yes	243	163 (67.08)	1 (0.41)	65 (26.75)	14 (5.76)		
No	3661	1939 (52.96)	55 (1.50)	1514 (41.35)	153 (4.18)		
Unknown	241	126 (52.28)	3 (1.24)	97 (40.25)	15 (6.22)		
Heart diseases						38.774^a^	< 0.0001
Yes	524	336 (64.12)	6 (1.15)	152 (29.01)	30 (5.73)		
No	3390	1772 (52.27)	49 (1.45)	1432 (42.24)	137 (4.04)		
Unknown	231	120 (51.95)	4 (1.73)	92 (39.83)	15 (6.49)		
Stroke						24.390^a^	< 0.001
Yes	313	195 (62.30)	2 (0.64)	92 (29.39)	24 (7.67)		
No	3604	1912 (53.05)	54 (1.50)	1491 (41.37)	147 (4.08)		
Unknown	228	121 (53.07)	3 (1.32)	93 (40.79)	11 (4.82)		

Notes: ^a^: Fisher exact test; missing data: education 2 (0.05%), marital status 47 (1.25%), smoking status 16 (0.39%), drinking status 33 (0.80%), regular exercise 94 (2.27%), diet of fresh fruit 10 (0.24%) and vegetables 8 (0.12%), and limited in activities because of health problems during the last six months 5 (0.12%)

The characteristics of the study population are provided in Table 1. There were 2228 (53.75%) clean fuel users, 59 (1.42%) participants never cooked, 1676 (1.42%) participants used biomass fuels for cooking, and 182 (4.39%) participants used other fuels for cooking among the total of 4145 older people.

According to χ^2^ tests, some characteristics were similar across the cooking fuels groups, but differences were found according to age, sex, education level, residence, household income, marital status, regular exercise, ventilation of the kitchen when cooking at home, diet of fresh fruit or vegetables, limited in activities because of health problems during the last 6 months, body mass index (BMI), and self-reported previous diseases.

Biomass fuel users were more likely to be observed in older people who were aged 65–84 years, were male and illiterate, lived in rural areas, had lower household incomes, were unmarried, had non-regular exercise, had no ventilation when cooking, consumed fresh fruit rarely or never, consumed vegetables occasionally, were limited in activities due to health problems during the last 6 months, were underweight, and had no self-reported previous diseases.

### Association of biomass fuel use for cooking and the risk of cognitive impairment

During 14,213.98 person-years of follow-up, a total of 432 incident cognitive impairment cases were observed. These included 216 cases, 10 cases, 175 cases, and 31 cases of participants that used clean fuels, never cooked, used biomass fuels, and used other fuels, respectively. Overall, the crude rate of cognitive impairment events was greater in the other three groups than in the clean fuel group (Table 2). In the unadjusted analysis, participants that reported never cooking, the use of biomass fuels, and the use of other fuels were all positively associated with an increased risk of cognitive impairment. Upon adjusting for the basic demographic characteristics, including age, sex, education, residence, household income, and marital status, the HR and 95% CI remained significant in the biomass fuel and other fuel user groups. In the multivariable-adjusted analysis, after adjusting for all of the covariates, the association between biomass fuel use and cognitive impairment risk had a slightly diminished magnitude, but was still significant (aHR: 1.27, 95% CI: 1.02–1.58). In addition, a greater risk of cognitive impairment was observed in the other fuel users compared with the clean fuel users (aHR: 1.82, 95% CI: 1.23–2.69).

**Table 2:** Association of cooking fuels with cognitive impairment in the univariate and multivariable models

Fuel	N events/Incidence rate (per 100 person-years)	Model 1		Model 2		Model 3	
		HR (95% CI)	*P*	HR (95% CI)	*P*	HR (95% CI)	*P*
Clean fuel	216 / 2.77	1 (reference)		1 (reference)		1 (reference)	
Never cooked in the home	10 / 5.97	3.04 (1.61, 5.74)	0.001	1.83 (0.97, 3.46)	0.064	1.63 (0.83, 3.22)	0.157
Biomass fuel	175 / 3.11	1.37 (1.12, 1.67)	0.002	1.30 (1.05, 1.60)	0.016	1.27 (1.02, 1.58)	0.030
Others	31 / 4.94	2.11 (1.45, 3.08)	0.000	1.88 (1.28, 2.77)	0.001	1.82 (1.23, 2.69)	0.003

Model 1 was a univariate model. The basic demographic characteristics were added in model 2, including age, sex, education, residence, household income, and marital status. All of the covariates in the model 3 were adjusted by adding smoking status, drinking status, regular exercise, ventilation of the kitchen when cooking at home, diet of fresh fruit and vegetables, limited in activities because of health problems during the last six months, BMI, and self-reported previous diseases based on model 2

The analysis was stratified by sex, education, residence, household income, smoking status, drinking status, fresh fruit, limited in activities because of health problems during the last six months, and self-reported previous diseases, and the significance of an interaction was tested by including a two-way interaction term in the final model. Sex, education, household income, drinking status, fresh fruit, limited in activities because of health problems during the last six months, and self-reported previous diseases had no significant group differences. Significant group differences were found in residence and smoking status in the association of fuel use for cooking with cognitive impairment in the multivariable-adjusted model (all *P* values for the interactions < 0.05). The risk of cognitive impairment was higher among the biomass fuel users who lived in rural areas (aHR: 1.44, 95% CI: 1.08–3.90) and never smoked (aHR: 1.33, 95% CI: 1.04–1.71). In addition, the risk of cognitive impairment was higher among the other fuel users who lived in rural areas (aHR: 2.19, 95% CI: 1.23–3.90) and smoked (aHR: 3.97, 95% CI: 1.84–8.58) (Table 3).

**Table 3:** Association of cooking fuel types with cognitive impairment stratified by participant characteristics

Subgroup	Biomass fuel Adjusted HR (95% CI)	Others Adjusted HR (95% CI)	***P***-value for interaction
All	1.27 (1.02, 1.58)	1.82 (1.23, 2.69)	
Sex			0.207
Male	1.26 (0.87, 1.83)	3.37 (1.72, 6.60)	
Female	1.16 (0.87, 2.30)	1.39 (0.84, 2.30)	
Education			0.604
No school	1.08 (0.83, 1.42)	1.66 (1.05, 2.62)	
1 year or more	1.54 (1.01, 5.53)	2.46 (1.10, 5.53)	
Residence			0.001
Urban	0.82 (0.55, 1.22)	1.53 (0.87, 2.68)	
Rural	1.44 (1.08, 3.90)	2.19 (1.23, 3.90)	
Household income CNY			0.197
< 10,000	1.52 (1.04, 2.21)	2.12 (1.05, 4.25)	
10,001–30,000	0.96 (0.61, 3.47)	1.67 (0.80, 3.47)	
> 30,000	0.99 (0.66, 1.51)	1.40 (0.71, 2.77)	
Smoking status			0.001
Non-smoker	1.33 (1.04, 1.71)	1.46 (0.91, 2.35)	
Smoker	0.66 (0.38, 8.58)	3.97 (1.84, 8.58)	
Drinking status			0.078
Non-drinker	1.19 (0.93, 1.53)	1.64 (1.06, 2.53)	
Drinker	0.96 (0.56, 9.41)	3.48 (1.28, 9.41)	
Regular exercise			0.876
Yes	1.28 (0.77, 2.12)	1.63 (0.78, 3.39)	
No	1.18 (0.91, 2.77)	1.72 (1.07, 2.77)	
Ventilation of the kitchen when cooking at home			0.826
no ventilation	0.74 (0.32, 1.69)	1.46 (0.42, 5.04)	
kitchen ventilation or fan	0.88 (0.55, 3.45)	1.07 (0.33, 3.45)	
by opening window	1.27 (0.93, 1.72)	1.86 (1.12, 3.10)	
unknown	0.56 (0.14, 2.25)	1.64 (0.27, 9.93)	
Fresh fruit			0.352
Almost or quite often	0.93 (0.63, 1.38)	1.24 (0.64, 2.41)	
Occasionally	1.19 (0.82, 4.27)	2.22 (1.15, 4.27)	
Rarely or never	1.68 (1.08, 2.61)	1.82 (0.75, 4.43)	
Limited in activities because of health problems during the last six months			0.449
Yes	0.78 (0.51, 1.20)	1.23 (0.59, 2.58)	
No	1.42 (1.09, 3.50)	2.18 (1.36, 3.50)	
Diabetes			0.006
Yes	3.33 (0.73, 15.19)	1.09 (0.18, 6.50)	
No	1.18 (0.93, 2.70)	1.76 (1.15, 2.70)	
Unknown	1.19 (0.33, 4.33)	3.00 (0.44, 20.68)	
Heart diseases			0.174
Yes	0.66 (0.26, 1.67)	1.99 (0.72, 5.46)	
No	1.25 (0.98, 2.55)	1.62 (1.03, 2.55)	
Unknown	1.02 (0.26, 4.03)	4.20 (0.48, 36.67)	
Stroke			0.077
Yes	0.82 (0.30, 2.20)	0.40 (0.08, 1.86)	
No	1.20 (0.95, 2.81)	1.84 (1.20, 2.81)	
Unknown	3.14 (0.67, 14.65)	101.82 (6.25, 1659.95)	

## Discussion

To our knowledge, the national longitudinal survey that examined the association of biomass fuel use for cooking with the risk of cognitive impairment among the elderly population aged 65 years and above in China has some limitations. We found that elderly people who reported that using biomass fuels for cooking had a significantly increased risk of cognitive impairment. In addition, the stratified results suggested that residence and smoking status modified the association of biomass fuel use with cognitive impairment in elderly Chinese people.

These results agreed with some studies that have reported that people who reported using biomass fuels for cooking had a significantly increased risk of cognitive impairment among adults. Krishnamoorthy et al. reported individuals who used biomass or kerosene as fuel were found to have two times more risk of having cognitive impairment according to a community-based cross-sectional study that included 295 adults [22]. This cohort study of the association between biomass fuel use for cooking and cognition among older adults was believed to be lacking, and Saenz et al. found that combustion of wood or coal fuels was associated with poorer cognitive performance in 13,023 Mexican adults over aged 50 using a cross-sectional study [12]. One study reported solid fuel users (coal, biomass charcoal/wood/ straw) had worse cognitive function that was evidenced by a lower global cognition score, mental health score, and episodic memory score in the Chinese middle-aged and older populations [23]. Cao et al. found that solid cooking fuel use was associated with a greater decline in the overall cognitive score, especially for the episodic memory of middle-aged and older participants according to follow-up studies that included 8397 participants [13]. Because biomass fuel use for cooking has a higher HAP, this study primarily examined the association of biomass fuel use with cooking and cognitive impairment. Additionally, potential influencing factors that included exercise, ventilation of the kitchen when cooking at home, diet, self-reported previous diseases, and other factors were controlled for in this study compared with the aforementioned studies. Additionally, the MMSE was used in this study to assess cognitive functioning that fully evaluates orientation, registration, attention and calculation ability, recall, and language ability, not just orientation, attention, and episodic memory, compared with most aforementioned studies. Finally, it was found that biomass fuel users had a higher risk of cognitive impairment (aHR: 1.27, 95% CI: 1.02–1.58) compared with participants who used clean fuels. Additionally, the results of this study also found the group differences due to residence and smoking status were significant based on the association of biomass fuel use with cognitive impairment. The risk of cognitive impairment was higher among the old people who lived in rural areas and never smoked. Saenz et al. also found that living in a more rural area was associated with lower scores across the cognitive function assessments [12]. This may have been because most of the participants in the rural areas reported using biomass as cooking fuels. This study was conducted in a large area that involved 23 research locations in 23 provinces in mainland China and the demographic characteristics (age, sex, education, residence, household income, and other factors), lifestyle (smoking status, drinking status, regular exercise, diet of fresh fruit and vegetables), and health status (BMI and self-reported previous diseases) were controlled for, which are related to cooking and cognitive function and affects the association of biomass fuel use with cognitive impairment. Based on these previous studies, this study further supplemented the results among elderly people and found a positive association between biomass fuel use and the risk of cognitive impairment to help develop intervention strategies focused on controlling the prevalence of cognitive impairment among elderly people in China, especially for older people living in rural areas.

The specific potential mechanism of cooking biomass fuel exposure-related cognitive impairment is unclear, but it may be linked to the PM released by the combustion of biomass fuels. The burning of biomass fuels causes high concentrations of PM [2, 3]. A large body of studies have suggested positive associations between PM and cognitive impairment. Ailshire et al. reported in 2014 [24], 2015 [25], and 2017 [26] that there was an inverse association between PM2.5 and cognitive function. Yuan et al. found PM_10_ was significantly associated with cognitive impairment (OR = 1.09, 95% CI: 1.02, 1.17) among Taiwanese older adults [27]. Salinas-Rodríguez et al. found that each 10 μg/m3 of increased ambient PM2.5 raised the odds of poorer cognitive function, including a three-word memory test and the number of valid animals named in a verbal fluency test [28]. The mechanisms involved may include brain oxidative stress, neuroinflammation, neurodegeneration [29], endothelial activation, and imbalanced autonomic nervous systems [30], all of which play key roles in the pathophysiology of brain states [29, 30].

According to the World Energy Outlook 2019 [1], China remains the world’s largest energy consumer in all scenarios, and the retrofitting capacity to co-fire with biomass plays an important role, particularly in China. This study found the proportion of biomass fuels users was 40.43% among Chinese older people. Additionally, biomass fuel users were more likely to be observed in older people who were illiterate, lived in rural areas, and had lower household incomes. These findings suggested that cooking energy alteration are still required, especially for people who are illiterate, live in rural areas, and have lower household incomes, to reduce the risk of adverse outcomes.

There are several limitations of this study. First, individual cognitive medicine use for improving cognition was not controlled for. However, the participants were free of cognitive impairments at the baseline, so this should not have altered the primary results of this prospective cohort study. Second, information regarding fuel stacking and stove types and other relevant indoor sources, such as heating and lighting, was lacking in the CLHLS survey. Hence, the effect of these factors could not be controlled for in this study. However, Cao et al. found that solid heating fuel use was associated with a greater decrease in the orientation and attention dimension [13]. Thus, this study may have overrated the effect of biomass cooking fuel on cognitive function. Third, education level has an important effect on the judgment of cognitive ability, while the elderly with lower educational levels account for a higher proportion of the older population in China. In addition, it is possible that participants with some hidden cognitive impairments could have answered inaccurately at the baseline, which may have caused a reversal causation. In order to ensure the quality of the survey, the project team had strictly and carefully trained investigators to conduct the household surveys to ensure the quality of the survey. All of the surveys were face-to-face interviews conducted at the participant’s home. If participant were illiterate, investigators helped them to complete the questionnaire. Fourth, the annual assessment of cognition and fuel exposure every year from 2014 to 2018 is preferred, however, the CLHLS study is a nationwide cohort study, and it is difficult for investigators to survey many times. Finally, this study did not measure an individual’s actual exposure dose of HAP from biomass fuel use, the secondary polluting fuel use that also accounts for high levels of HAP [4] and the lifetime exposure to HAP. Thus, further studies should include a more objective and exhaustive assessment of individual exposure to indoor air pollution and fuel use to confirm these findings.

## Conclusions

The results of this nationwide prospective cohort study suggested that biomass fuel used for cooking was associated with cognitive impairment, as defined by MMSE, in a population-based study of elderly in China. In addition, the risk was higher among older people who lived in rural areas and never smoked. These findings suggested that altering the use of biomass fuels to green and clean energy is required to decrease the risk of cognitive impairment among Chinese older people, especially those who live in rural areas.

## Supplementary Information

**Additional file 1: Table S1.** Association of cooking fuels with cognitive impairment in the univariate and multivariable models among excluded 3047 participants. **Table S2.** Association of cooking fuels with cognitive impairment in the univariate and multivariable models among 2933 participants after excluded 1212 participants who died in the follow-up survey.

## Data Availability

Data are from the Chinese Longitudinal Healthy Longevity Survey 2011–2018 which is a public, open access repository (https://opendata.pku.edu.cn).

## Abbreviations

CLHLS: Chinese Longitudinal Healthy Longevity Survey
MMSE: Mini-Mental State Examination
HAP: Household air pollution
PM: Particulate matter
LPG: Liquefied petroleum gas
BMI: Body mass index
SDs: Standard deviations
IR: Incidence rates
HRs: Hazard ratios
CIs: Confidence intervals
