# Supplementary material for: Association between biomass fuel use and the risk of cognitive impairment among older populations in China: a population-based cohort study
Source: Environ Health. 2021 Feb 24;20:21. doi: 10.1186/s12940-021-00706-1 (PMC7905553; doi:10.1186/s12940-021-00706-1)
Supplement: Supplementary file 1 — Additional file 1: Table S1. Association of cooking fuels with cognitive impairment in the univariate and multivariable models among excluded 3047 participants. Table S2. Association of cooking fuels with cognitive impairment in the univariate and multivariable models among 2933 participants after excluded 1212 participants who died in the follow-up survey. [file 12940_2021_706_MOESM1_ESM.docx]

**Supplemental table 1 Association of cooking fuels with cognitive impairment in the univariate and multivariable models among excluded 3047 participants**

| Fuel | N events/Incidence rate (per 100 person-years) | Model 1 | |  | Model 2 | |  | Model 3 | |
| --- | --- | --- | --- | --- | --- | --- | --- | --- | --- |
|  |  | HR (95% CI) | *P* |  | HR (95% CI) | *P* |  | HR (95% CI) | *P* |
| Clean fuel | 75 /1.69 | 1 (reference) |  |  | 1 (reference) |  |  | 1 (reference) |  |
| Never cooked in the home | 7 /3.47 | 1.97 (0.90 ,4.28) | 0.088 |  | 1.36 (0.61 ,3.02) | 0.453 |  | 1.31 (0.52 ,3.29) | 0.570 |
| Biomass fuel | 103 /3.43 | 1.49 (1.09 ,2.02) | 0.011 |  | 1.12 (0.80 ,1.58) | 0.498 |  | 0.82 (0.57 ,1.17) | 0.266 |
| Others | 6 /1.62 | 0.79 (0.34 ,1.82) | 0.578 |  | 0.57 (0.24 ,1.34) | 0.195 |  | 0.77 (0.32 ,1.85) | 0.558 |

Model 1 was a univariate model. The basic demographic characteristics were added in model 2, including age, sex, education, residence, household income, and marital status. All of the covariates in the model 3 were adjusted by adding smoking status, drinking status, regular exercise, ventilation of the kitchen when cooking at home, diet of fresh fruit and vegetables, limited in activities because of health problems during the last six months, BMI, and self-reported previous diseases based on model 2.

**Supplemental table 2 Association of cooking fuels with cognitive impairment in the univariate and multivariable models among 2933 participants after excluded 1212 participants who died in the follow-up survey**

| Fuel | N events/Incidence rate (per 100 person-years) | Model 1 | |  | Model 2 | |  | Model 3 | |
| --- | --- | --- | --- | --- | --- | --- | --- | --- | --- |
|  |  | HR (95% CI) | *P* |  | HR (95% CI) | *P* |  | HR (95% CI) | *P* |
| Clean fuel | 216 /3.38 | 1 (reference) |  |  | 1 (reference) |  |  | 1 (reference) |  |
| Never cooked in the home | 10 /8.65 | 3.04 (1.61 ,5.73) | 0.001 |  | 1.79 (0.95 ,3.39) | 0.073 |  | 1.61 (0.82 ,3.17) | 0.169 |
| Biomass fuel | 175 /3.72 | 1.35 (1.10 ,1.65) | 0.003 |  | 1.28 (1.04 ,1.58) | 0.021 |  | 1.28 (1.03 ,1.59) | 0.027 |
| Others | 31 /6.44 | 2.16 (1.48 ,3.15) | 0.000 |  | 1.87 (1.27 ,2.75) | 0.002 |  | 1.81 (1.22 ,2.69) | 0.003 |

Model 1 was a univariate model. The basic demographic characteristics were added in model 2, including age, sex, education, residence, household income, and marital status. All of the covariates in the model 3 were adjusted by adding smoking status, drinking status, regular exercise, ventilation of the kitchen when cooking at home, diet of fresh fruit and vegetables, limited in activities because of health problems during the last six months, BMI, and self-reported previous diseases based on model 2.
